# Ageing population and society: a scientometric analysis

**DOI:** 10.1007/s11135-022-01509-3

**Published:** 2022-08-25

**Authors:** Muhammad N. Mahmood, Subas P. Dhakal

**Affiliations:** 1 Deakin University (Waterfront Campus), 1 Gheringhap St, 3220 Geelong, VIC Australia; 2 University of New England (UNE), Trevenna Road, 2351 Armidale, NSW Australia

**Keywords:** Ageing agenda, Betweenness centrality, Gephi, North-South collaboration, Policy innovation, Sustainable development, VOSviewer

## Abstract

The ageing population and society (APS) nexus is one of the key grand challenges of this millennium. And yet, the systematic analysis of scholarly literature on the APS nexus has remained under the radar. This study responds to this gap and employs a quantitative approach through a scientometric analysis of literature on the APS nexus to inform policy discussions and guide future research directions. This study adopts quantitative scientometric methods to examine the APS literature (n = 566) between 2011 and 2020 found in the Scopus database. The analysis reveals key research topics and recognizes the most important articles, authors, publication outlets, institutions, and countries in the field. The findings indicate that while issues such as ageing population, gender, quality of life, and socio-economic aspects of ageing have received significant interest, social exclusion of older adults, age diversity, social policy, and the eldercare workforce have received less attention. As challenges associated with the APS nexus will continue to gain currency in the future, this paper discusses the implications of the findings on (a) future research direction and (b) north-south research collaboration. The analysis shown in this paper should be of interest to scholars and policymakers interested in addressing the challenges associated with the APS nexus.

## Introduction

Ageing population is a transformative social phenomenon that increases the proportion of older adults within the total population that affects or will affect both developed and developing societies (Dobrescu and Smith 2016; ILO, 2009). Although the idea of ageing is context-dependent and can mean many things in many societies (see Dhakal et al. 2022; Powell and Hendricks 2009), the World Health Organisation (WHO) has adopted a narrative around healthy ageing – developing and maintaining the functional ability that enables the wellbeing of elderly that warrants societies to:) change the way we think about aging and elderly, (ii) create age-friendly environments, (iii) align health systems to the needs of the elderly, and (iv) develop strategies for long-term care (WHO, 2019, p. 4).

The elderly demographic bracket includes people aged 65 years old and over (65+) and the United Nations Department of Economic and Social Affairs (UNDESA) predicts that one in six people globally will be 65 + years old by 2050 (UNDESA, 2019). On the one hand, this demographic shift represents progress in medical sciences and socio-economic development across the globe. On the other, as one of the most significant demographic phenomena that is transforming societies and challenging existing institutional solutions (Ervik & Lindén, 2013), the ageing population and society (APS) nexus has gained currency as a significant policy innovation issue within the United Nation’s 17 Sustainable Development Goals (SDGs) (see Álvarez-García et al. 2019).

Conliffe et al. (2018, p. 6) highlight that “policy innovation encompasses both the “what” (the policy instrument or artifact that gets created) and the “how” (the processes and tools by which policy is created and developed). Policy innovation research is often driven by market forces and influenced by political influences and public opinions (Burstein 2003; Pawson and Wong 2013). Consequently, it has been argued that the ageing agenda to minimise socio-economic costs and maximise the wellbeing of the elderly has not been a priority in the west (Walker 2018). This paper contends that a scientometric analysis of the APS-related literature can capture existing and emerging research trends to generate valuable policy insights and guide future research directions.

This paper is structured in four parts, with the following section providing a background to scientometrics in the context of the ageing agenda. The subsequent sections describe methods and findings and discuss implications before concluding remarks.

## Scientometrics and the ageing population and society nexus

A quick search of “scientometric analysis” in Google Scholar yielded 37,000 results in July 2021. When the search was limited between 2011 and the present, it produced 18,700 results, indicating a phenomenal growth in the past decade. Scientometric techniques can unravel factors that drive knowledge advancements, such as academic institutions, individual researchers, and research clusters. Chellappandi and Vijayakumar (2018) describe scientometrics as an approach that: “analyses the quantitative aspects of the production, dissemination, and use of scientific information to achieve a better understanding of the mechanisms of scientific research as a social activity” (p. 6). Hood and Wilson (2001) posited that scientometric analysis as a method could overcome the challenges associated with manual literature reviews by processing a large amount of bibliometric data, thereby assisting researchers in exploring and reviewing literature by identifying conceptual linkages. Scientometrics is often regarded as a tool for generic information visualization (Hook, 2007) because it mainly facilitates domain mapping and graphical representations of literature through subjective visualizations (Hook & Borner, 2005). While Chen et al. (2011) pointed out that the method is suited for gauging the social impact of research outputs, Mingers and Leydesdorff (2015) suggested that scientometrics is helpful in analysing citations in the literature. The technique can also be applied to facilitate the mapping of knowledge structure and advancements in any field of research (Zhong et al. 2019; Park and Park 2018). For instance, one of the most critical features of scientometric techniques happens to be the aspect of graphical representation of bibliometric data, enabling the researchers to express a global view of the subject while retaining the salient characteristics of the domain, like its dynamics, structural details, and establishing the research outcomes of most-cited authors and papers (Hook & Borner, 2005).

Given that the APS nexus has emerged as a priority for countries worldwide, scientometric analysis can unravel the factors that drive knowledge advancements on the ageing agenda, such as vital academic institutions, individual researchers, and research clusters. Béland (2016) highlighted the importance of adopting rigorous methodologies to inform future research directions. Several scientometric analyses have been carried out on ageing population as a transformative social phenomenon. For instance, (a) Álvarez-García et al. (2019) examined the literature on the issue of older adults and digital society, (b) Dominko and Verbič (2019) explored the topic of subjective wellbeing among the elderly, (c) Mahmood & Dhakal (2022) examined the aging-related studies in selected countries of the Asia Pacific region, (d) Müller et al. (2016) carried out a bibliometric analysis on the theme of physical activity and ageing, and (e) Xiang et al. (2020) examined the theme of age-friendly cities and communities. However, studies on the APS nexus remain fragmented, and this paper responds to this gap. This paper has the following two research objectives:

1. To identify APS-related research topics, main keywords, thematic clusters, and journals.
2. To generate insights on the APS-related policies and future research directions.

## Data and methodology

Drawing on Hosseini et al. (2018) and Mahmood & Dhakal (2022), this research utilises quantitative scientometric methods and adopts a four-staged process: (a) selection of software, (b) selection of database, (c) screening of research outputs, and (d) network analysis and visualization. First, the software selection was based on multiple criteria: cost, diagnostic features, capabilities of investigators, and user-friendliness. This study utilised *Microsoft Excel* 2018; *VOSviewer* 1.6.1.13, and *Gephi* 0.9.2. *Microsoft Excel* enables scientometric analysis by sorting and creating data visualisations (Sweileh et al., 2017). *VOSviewer* is free software that provides fundamental features to explore scientometric linkages and networks (Hilal et al., 2019; van Eck & Waltman, 2019). *Gephi* facilitates the exploration and manipulation of graphical data and allows insightful quantitative analysis from information networks (Wuni et al. 2019; Hasan et al. 2021). Three of the software mentioned above facilitated quantitative meta-analysis to produce top research outlets, co-occurrence of keywords mapping, research collaboration networks, prominent institutions collaboration networks, and country collaboration networks.

**Fig. 1:**
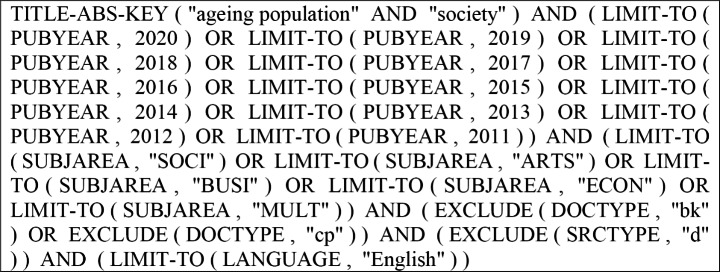
The complete search code used

Second, selecting a literature database for accessing the bibliometric data was based on the two criteria: access and coverage. Scopus and Web of Science are two common databases used in the scientometric analysis. Nonetheless, sourcing information from multiple sources remains tricky due to the complexities associated with checking outputs duplicities. Scopus database was selected in order to retrieve the literature because of the scope and magnitude of the coverage, efficient indexing process, and recency of publications (Sankar 2019; Hasan et al. 2021). Third, an explicit and reproducible search code used in selecting literature is shown in Fig. 1. The search was conducted using the keywords “ageing population” AND “society” in the Scopus database. The search was limited to the 2011–2020 period and was not confined to just one country or region. Based on the initial search, 644 publications were identified. Based on literature screening, non-journal research outputs, such as trade journals, edited books, and conference proceedings were excluded from the analysis. A total of 566 journal articles were selected for the analysis. The fourth and final stage involved creating maps to examine the research trends in the past decade and identify key journal outlets, core research themes, influential scholars, organisations, and countries.

## Results

### Research trend (2011–2020)

**Fig. 2:**
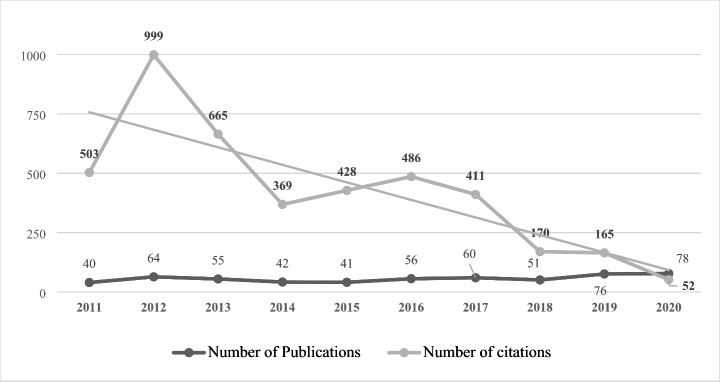
Research outputs trend on the APS nexus (2011–2020)

Research outputs show steady growth over the last ten years. Figure 2 shows that 242 articles (43%) were published between 2011 and 2015, and 321 articles (57%) were published between 2016 and 2020. The year 2019 featured considerably more articles than any other year. Although the justification for this peak was investigated, a definitive reason was unclear and could not be determined. The findings demonstrate that the pattern of research outputs did not show a continuous growth or decline in this research topic. Publication count ranged between 40 and 60. The overall number of citations for papers published in 2012 was the highest, most likely because they have been available for a more extended period. Additionally, journal articles published in 2011, 2013, and 2016 had a significant number of citations (n ≥ 450), indicating the importance of the APS-related research published during those years. In terms of specific papers, Milligan et al. (2011) had the most citations (n = 109), followed by Arai et al. (2012) and Culhane et al. (2013), with 106 and 81 citations, respectively.

### Top outlets

According to the initial sample statistics based on the scopus database, Table 1 illustrates the top 10 ranks of contributing journals. The journal ‘Ageing and Society’ published the most journal articles (n = 45), followed by ‘Sustainability Switzerland’ (n = 24), ‘Social Science and Medicine’ (n = 14), and ‘Journal of Aging and Social Policy’ (n = 12). A good match between the journal and the manuscript may improve the odds of the manuscript being published; thus, identifying key outlets may be very helpful to authors when it comes to selecting suitable outlets for publishing their work (Chen 2004). The focused research themes of the top outlets are also presented in Table 1. As expected, the most common themes were ageing society, ageing in place, long-term ageing care, social engagement, and other issues related to the ageing agenda.

**Table 1:** Top contributing journals on APS related themes between 2011 and 2020

Rank	Journal	No. of Articles	Focus Areas
1	Ageing and Society	45	Ageing society, older adults, social participation, life transitions
2	Sustainability Switzerland	24	Ageing society, population ageing, social sustainability, sustainable development
3	Social Science and Medicine	14	Productive ageing, social engagement, gender, Asia
4	Journal of Aging and Social Policy	12	Ageing in place, long-term care, age-friendly, community-based services
5	Plos One	8	Aged, hearing loss, human experiment, clinical study
5	Technological Forecasting and Social Change	8	Technology, ageing society, older individuals, senior care
6	Ageing International	7	Population ageing, long-term care, social work, older adults
6	Educational Gerontology	7	Human experiment, adult, ageing, residential care
6	Journal of Rural Studies	7	Rural ageing, community development, older adults, private sector businesses
7	Anthrozoos	6	Human-animal interaction, health, ageing, older adults
7	Geriatrics and Gerontology International	6	Elderly, ageing, early functional loss, dementia
8	Social Indicators Research	5	Life expectancy, elderly people, ageing society, living arrangement
9	Health and Social Care in The Community	4	Palliative care, community rehabilitation, older people, service satisfaction
10	Cities	3	Ageing in place, older adults, public acceptability, por-social value orientations and social norms
10	Generations	3	Palliative care, public health, eldercare workforce, healthcare technology
10	Gerontology and Geriatrics Education	3	Age diversity, age inclusivity, higher education, ageing society
10	Health and Place	3	Ageing, care, rural environment, social exclusion
10	Iatss Research	3	Technical innovation, transport and communication analogy, ageing society, internet of things
10	Social Policy and Administration	3	Social policy, immigration policy, population ageing, Asia
10	System Dynamics Review	3	Ageing population, education policy, population modelling, decision support system

### Research Focus (co-occurrence of keywords analysis)

The core subject matter and range of research themes contained in publications can be identified through the keywords. Similarly, a well-connected network of relevant keywords enables a practical comprehension of the connections, trends, and intellectual structure of the areas covered by scientific knowledge creation throughout time (van Eck & Waltman, 2019). In addition, the analysis of the co-occurrence of keywords represented through network maps generates insightful observations in the development of a field of research (see Khaldi and Prado-Gascó 2021).

**Fig. 3:**
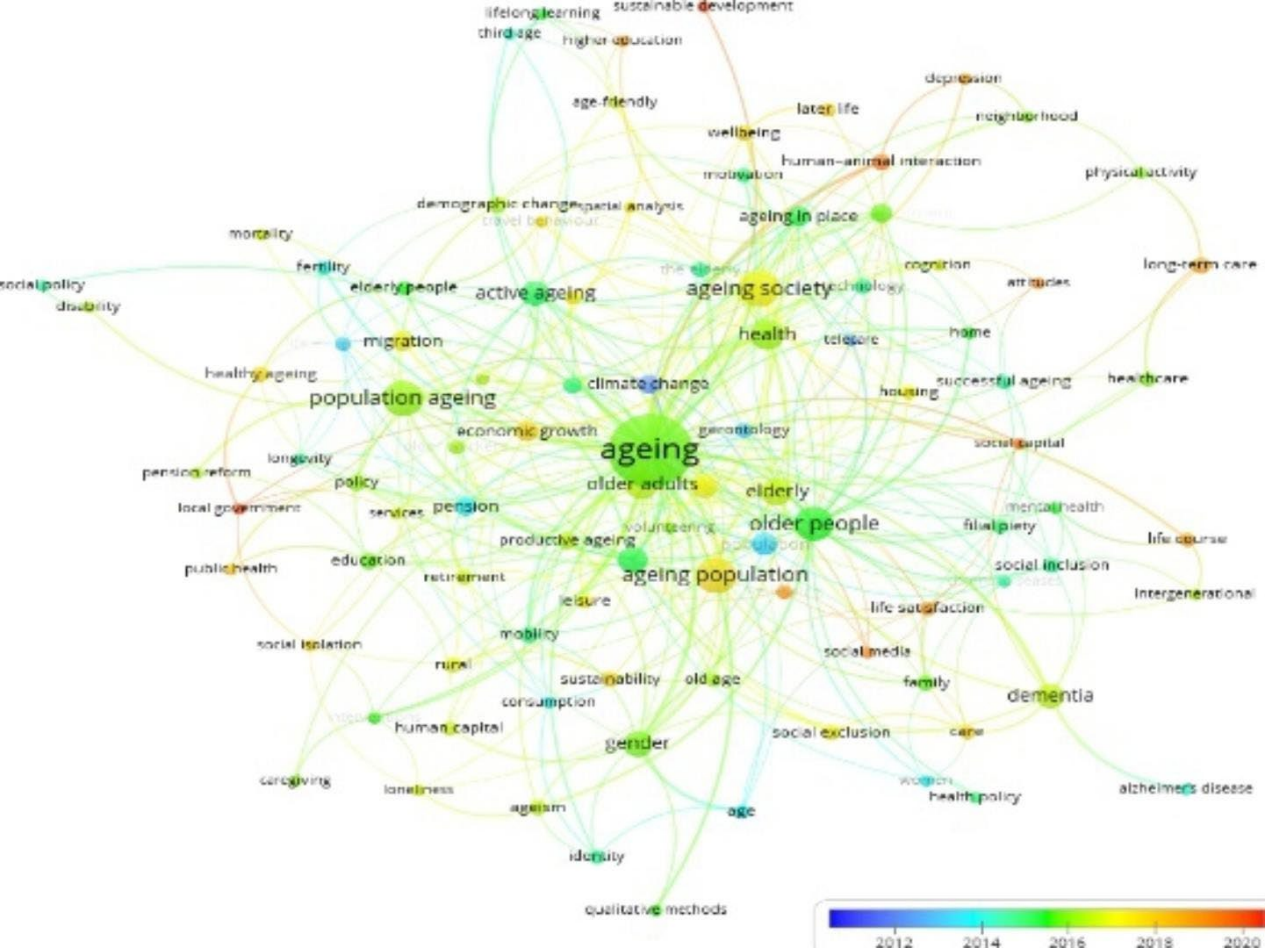
Co-occurrence map of APS related research themes

In *VOSviewer*, a full counting option was used to extract 1629 author keywords from the Scopus dataset. The default value was set at three for the minimal number of occurrences, and a total of 110 keywords satisfied the threshold requirement. After undertaking co-occurrence of keywords analysis, identical items, such as ‘ageing’ and ‘aging’; ‘ageing in place’ and ‘aging in place’; ‘ageing population’ and ‘aging population’; ‘pension’ and ‘pensions’; ‘older adults’ and ‘older adult’; ‘travel behaviour’ and ‘travel behavior’ were merged. Additionally, after excluding generic terms, such as Australia, China, Hong Kong, Japan, Poland, Taiwan, the UK, and the USA, 98 research themes connected through 324 links with total link strength of 475 (Fig. 3) were obtained. The depth and weight of the connections between keywords indicate the number of articles in which the keywords appear in combination.

The co-occurrence map generated in *VOSviewer* was then submitted to *Gephi* to determine each research theme’s degree of centrality value. The degree centrality values are used to rank the main research themes in Table 2. A greater degree centrality score indicates that the research theme is more influential (Opsahl et al. 2010). If two or more research themes are equivalent in their degree centrality, the one with the most significant betweenness centrality is regarded to have the most impact. The greater the betweenness centrality value, the higher the chance of a node in the network being on the shortest path between nodes (Gephi, 2017; Glynatsi and Knight 2021). The most important research themes and their connectedness, as demonstrated in Fig. 3, revealed several interesting findings, including the ranking of research themes, reflecting gaps within the literature. The analysis revealed a primary concentration on some research areas, while less emphasis was placed on others. For example, ageing, older people, health, demography, older adults, gender, population ageing, ageing society, active ageing, and quality of life have been key influential themes. In contrast, less attention has been paid to sustainable development, social policy, public health, health policy, disability, caregiving, and Alzheimer’s disease.

**Table 2:** Research themes on the APS nexus

Research Themes	Degree centrality	Betweeness centrality	Relative Importance
Ageing	125.00	130.38	1
Older people	41.00	246.88	2
Health	37.00	138.33	3
Demography	25.00	85.79	4
Older adults	22.00	91.65	5
Gender	22.00	46.81	6
Population ageing	22.00	34.85	7
Ageing society	20.00	20.00	8
Active ageing	20.00	19.32	9
Quality of life	18.00	68.45	10
Elderly	17.00	115.04	11
Population	17.00	17.58	12
Climate change	17.00	3.80	13
Built environment	14.00	19.13	14
Ageing population	14.00	11.78	15
Technology	13.00	16.15	16
Dementia	12.00	47.17	17
Life expectancy	12.00	45.60	18
Ageing in place	12.00	37.01	19
Economic growth	12.00	32.78	20
Pension	12.00	23.58	21
Wellbeing	12.00	3.13	22
Social inclusion	11.00	5.96	23
Retirement	11.00	4.43	24
Policy	10.00	137.57	25
Life satisfaction	10.00	23.44	26
Fertility	10.00	20.00	27
Migration	10.00	18.00	28
Human-animal interaction	10.00	12.00	29
Education	9.00	44.47	30
Productive ageing	8.00	93.43	31
Mobility	8.00	59.77	32
Filial piety	8.00	20.33	33
Family	8.00	14.12	34
Cognition	8.00	5.96	35
Chronic diseases	8.00	4.00	36
Demographic transition	8.00	3.57	37
Housing	8.00	2.38	38
Accessibility	8.00	1.21	39
Telecare	7.00	27.50	40
Interventions	7.00	20.00	41
Gerontology	7.00	16.76	42
Volunteering	7.00	14.43	43
Motivation	7.00	9.33	44
Longevity	7.00	4.27	45
Care	7.00	3.34	46
Age	7.00	2.52	47
Successful ageing	7.00	2.00	48
Demographic change	7.00	1.59	49
Mental health	6.00	28.25	50
Healthy ageing	6.00	14.39	51
Healthcare	6.00	10.23	52
Age-friendly	6.00	9.00	53
Home	6.00	5.66	54
Ageism	6.00	5.27	55
The elderly	6.00	4.33	56
Third age	6.00	3.76	57
Travel behaviour	6.00	2.37	58
Identity	6.00	2.16	59
Services	6.00	1.80	60
Well-being	6.00	1.28	61
Social isolation	6.00	0.40	62
Neighbourhood	5.00	11.00	63
Rural	5.00	8.75	64
Local government	5.00	8.56	65
Old age	5.00	7.27	66
Social media	5.00	4.73	67
Leisure	5.00	4.02	68
Consumption	5.00	3.27	69
Spatial analysis	5.00	2.72	70
Women	5.00	2.18	71
Life-long learning	5.00	1.51	72
Loneliness	5.00	0.50	73
Long-term care	4.00	4.50	74
ICT	4.00	3.10	75
Elderly people	4.00	3.00	76
Higher education	4.00	2.00	77
Life course	4.00	1.32	78
Human capital	4.00	2.87	79
Older workers	4.00	1.65	80
Social capital	4.00	1.32	81
Social exclusion	4.00	0.80	82
Depression	3.00	0.83	83
Attitudes	3.0	0.00	84
Intergenerational	3.00	0.00	85
Later life	3.00	0.00	86
Mortality	3.00	0.00	87
Pension reform	3.00	0.00	88
Physical activity	3.00	0.00	89
Qualitative methods	3.00	0.00	90
Sustainability	3.00	0.00	91
Alzheimer’s disease	2.00	0.00	92
Caregiving	2.00	0.00	93
Disability	2.00	0.00	94
Health policy	2.00	0.00	95
Public health	2.00	0.00	96
Social policy	2.00	0.00	97
Sustainable development	1.00	0.00	98

In addition, some of the established and emerging themes were identified based on the co-occurrence network of author keywords analysis using the Overlay Visualisation functionally of *VOSviewer*, as shown in Fig. 3. On the one hand, some of the key themes between 2011 and 2014 were climate change, telecare, population, life expectancy, fertility, social inclusion, longevity, gerontology, consumption, women, pension, and Alzheimer’s. On the other, some of the key themes that emerged between 2018 and 2020 were sustainable development, human-animal interaction, long-term care, depression, social capital, social media, attitudes, life satisfaction, wellbeing, public health, local government, and higher education.

The results also indicated that “ageing” has the highest degree centrality value. This finding is expected given that the researchers have used ageing as one of the keywords in the search. The research theme with the second-largest degree centrality value (41) is older people. Similar research themes include elderly, older adults, and elderly people. Older people are recognised as one of the key contributors to population ageing. According to Land and Lamb (2008), “Population aging refers to changes in the age composition of a population such that there is an increase in the proportion of older persons” (p. 90). The notion can partially explain why older people and population ageing have second- and seventh-largest degree centrality values, respectively.

In contrast, research themes with the lowest centrality score were sustainable development, followed by social policy, public health, health policy, and disability, indicating the disintegration of these research themes from the main body of literature. These findings support the sentiment that studies linking the ageing population, and sustainable development remain scarce (Kudo et al. 2015). Given the UN SDGs came into effect in 2015, the finding is self-explanatory. The fragmentation of sustainable development and policy innovation on the ageing agenda has been highlighted in the literature (Liu et al. 2020; Sobczak et al. 2020; Malik and Mikołajczak 2019). As a collective intervention aimed at enhancing access to adequate and secure livelihoods, income, and social services, policy innovation can be a critical component of developing an integrated strategy for sustainable development due to its inherent cross-cutting nature (Johansson et al. 2011).

### Scientific collaboration networks (co-authorship analysis)

**Fig. 4:**
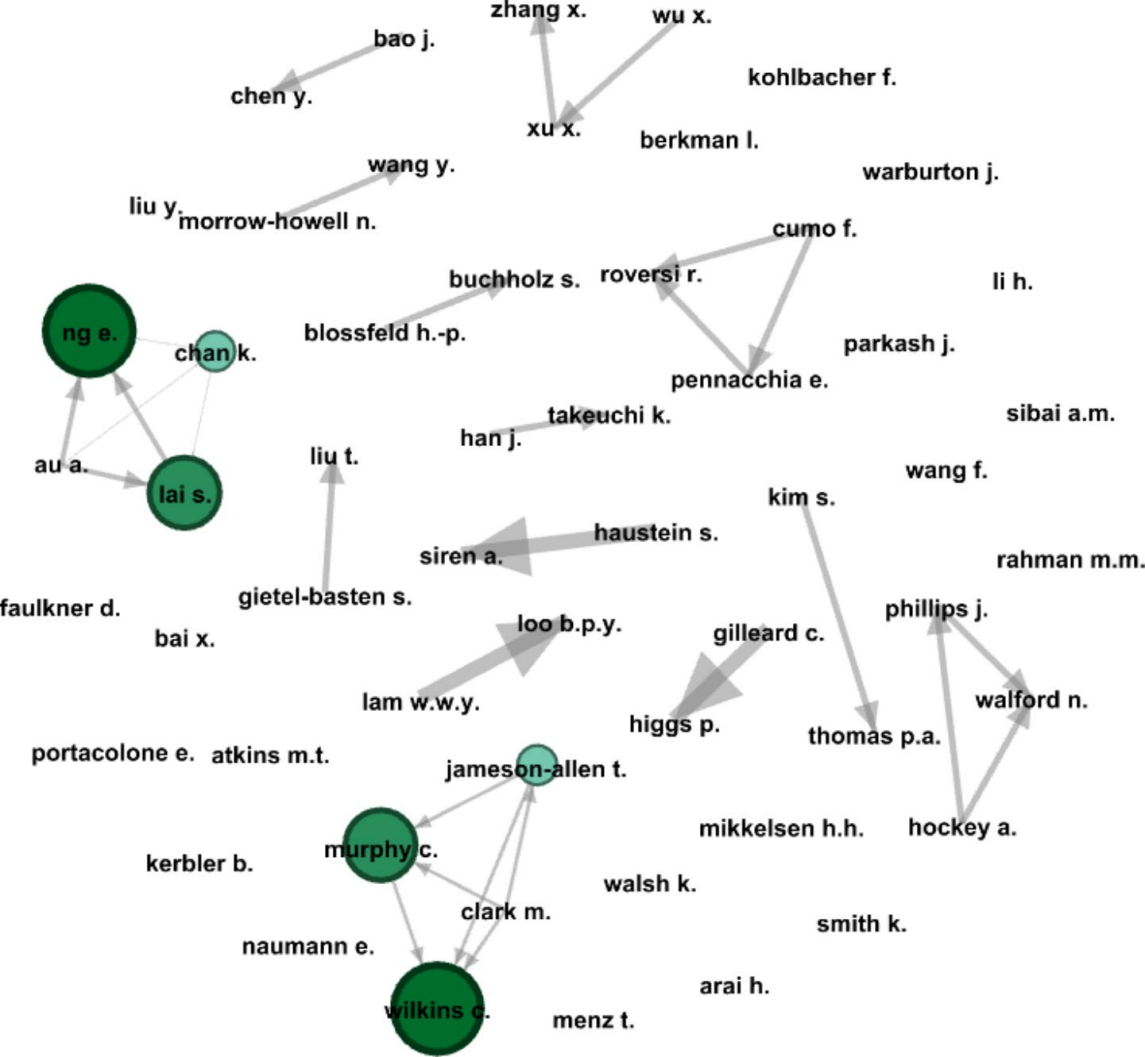
Research collaboration network on the APS nexus

The analysis examined influential investigators, leading organisations, and countries where the studies were carried out. Awareness of a well-connected and collaborative research team in any particular area improves academic communication and productivity while enhancing accessibility to funding, cross-fertilisation of ideas and specialties (Ding 2011). According to Glänzel and Schubert (2005), collaborative research results are expected to be published in high-impact journals with more citations. In *VOSviewer*, for the co-authorship analysis, “authors” was set as the unit of analysis and ‘fractional counting’ for the counting method. To create a manageable network, two criteria were established: each author must have at least two articles and a minimum of five citations. After applying the criteria, 55 out of 1451 identified authors met the requirements. *VOSviewer’s* network map showed 55 authors connected through 29 links in 14 clusters. For calculating the authority score, the network map was then imported to *Gephi*. Figure 4 depicts the HITS algorithm’s network map, where darker colours indicate more prominent nodes with better authority scores. According to Khokhar (2015), the HITS algorithm ranks the authors in the network depending on their authority scores. A higher authority value signifies influential and resourceful sources of information in a network, which may be widespread and immensely collaborative with a plethora of contributions.

The two major collaborative networks headed by Ng E. (Hong Kong Polytechnic University) and Wilkins C. (Sporting Memories Network, UK) are shown in Fig. 4. Several emerging collaborations were observed in the network, such as Phillips J., Walford N., and Hockey A.; Cumo F., Roversi R., and Pennacchia E. These collaborative networks underline the continuous interchange and knowledge generation via joint research. Figure 4 also highlights that many authors who are not members of the established collaborative networks also demonstrate solitary research activities.

**Fig. 5:**
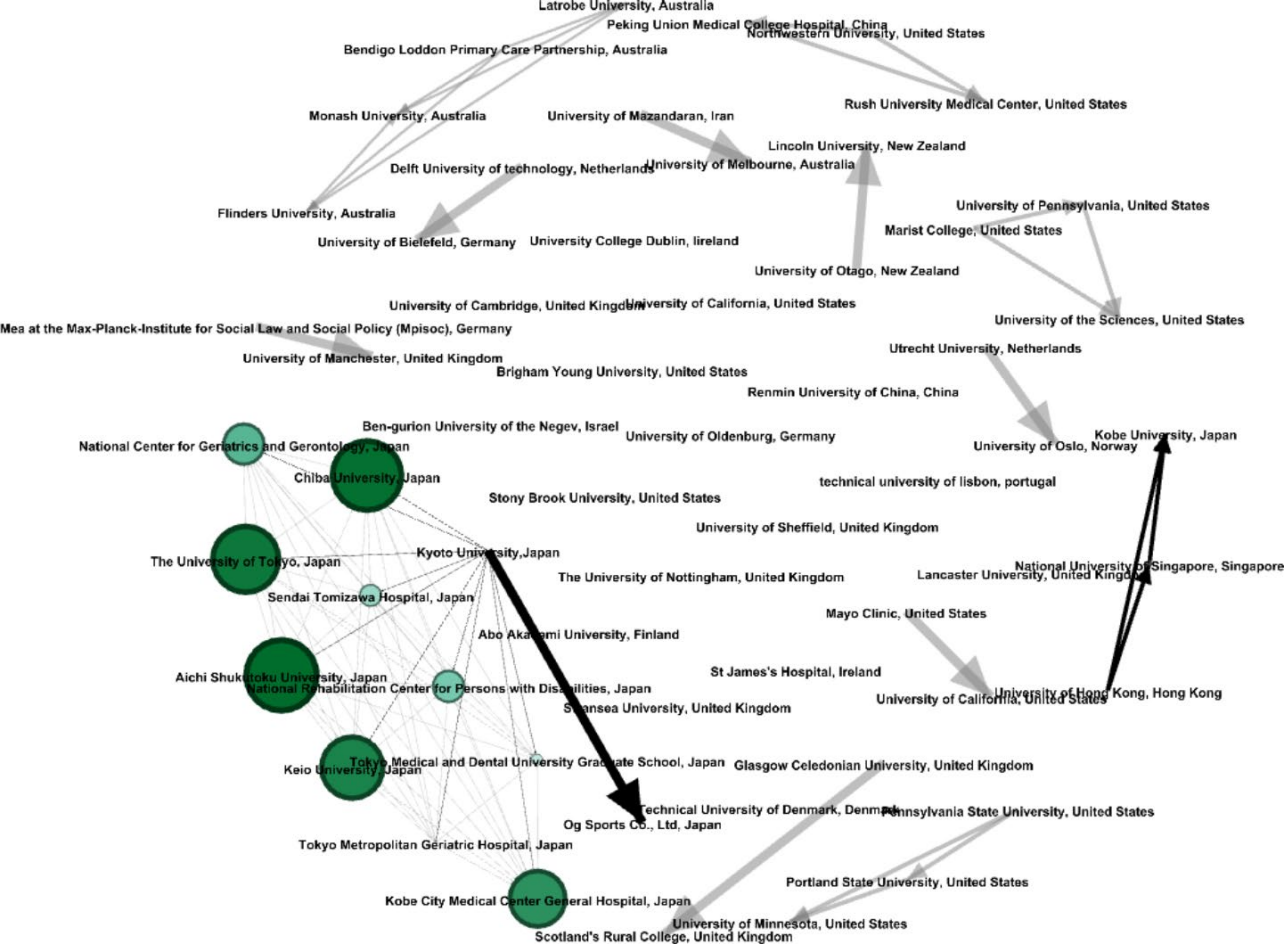
Prominent institutions conducting the APS related research

Next, using *VOSviewer*, bibliometric data were analysed by selecting “co-authorship,“ “organisation,“ and “fractional counting” as the type of analysis, unit of analysis, and counting technique, respectively. Alongside highlighting the collaboration amongst individual investigators, the co-authorship analysis enables the identification of collaboration between institutions through visual networks. Essentially, it represents a network of institutions motivated by deeper interests and funded with more significant investments in specific domains (Ding 2011). The dataset included 202 institutions and organisations, but only 34 were included in the network map since they had at least one document and at least 20 citations. *VOSviewer’s* network map was then transferred to Gephi, where the HITS algorithm was used to visualise, using darker colours to represent nodes with greater hub scores (see Fig. 5). HITS algorithm captures the hub scores for institutions, reflecting the volume and credibility of specific nodes (Khokhar 2015). Higher hub scores signify credible and noteworthy institutions in the domain field under research and act as benchmarks for further reference by other institutions.

In Japan, *Aichi Shukutoku University* is the top institution for research on Ageing population and society, followed by *Chiba University, The University of Tokyo*, and *Keio University*. Figure 5 also shows collaborations of several institutions, such as *Kobe City Medical Centre General Hospital*, *National Centre for Geriatrics and Gerontology*, National *Rehabilitation Centre for Persons with Disabilities*, Sendai *Tomizawa Hospital*, *Tokyo Medical and Dental University Graduate School*, *Kyoto University*, and *Tokyo Metropolitan Geriatric Hospital* along with the universities mentioned above in Japan. The figure explains the dominance of Japan as one of the most influential countries in the area of research on the APS nexus. In the context of Australia, one interconnection can be observed between *Flinders University*, *Monash University*, *Latrobe University*, and *Bendigo Loddon Primary Care Partnership*. In the UK, several institutions are active in this research area; however, only one strong collaboration is observed between *Glasgow Caledonian University* and *Scotland’s Rural College*. In the US, three collaborations are marked (e.g., among Pennsylvania State University, the University of Minnesota, and Portland State University; among Marist College, University of the Sciences, and the University of Pennsylvania; between Mayo Clinic and the University of California). In New Zealand, one interconnection between the University of Otago and Lincoln University is observed. Additionally, Fig. 5 also reveals interlinks between different institutions of various countries (e.g., the *Delft University of Technology* and *University of Bielefeld*; *Utrecht University* and *University of Oslo*; *Kobe University*, *National University of Singapore* and *Lancaster University*; *Peking Union Medical College Hospital*, *Rush University Medical Centre* and *Northwestern University*; the *University of Melbourne* and *University of Mazandaran; University of Manchester* and *Mea at the Max-Planck-Institute for Social Law and Social Policy*).

Beyond identifying the institutions, the co-authorship analysis also gauged the most influential countries concerning the specialisation of subjects and their associated research based on the countries and the aspect of collaboration between nations. Using *VOSviewer*, the bibliometric data were analysed by selecting “co-authorship,“ “countries,“ and “fractional counting” as the type of analysis, unit of analysis, and counting technique, respectively. Only 19 of the 67 countries in the dataset met the requirement of a minimum of 10 papers and five citations.

**Fig. 6:**
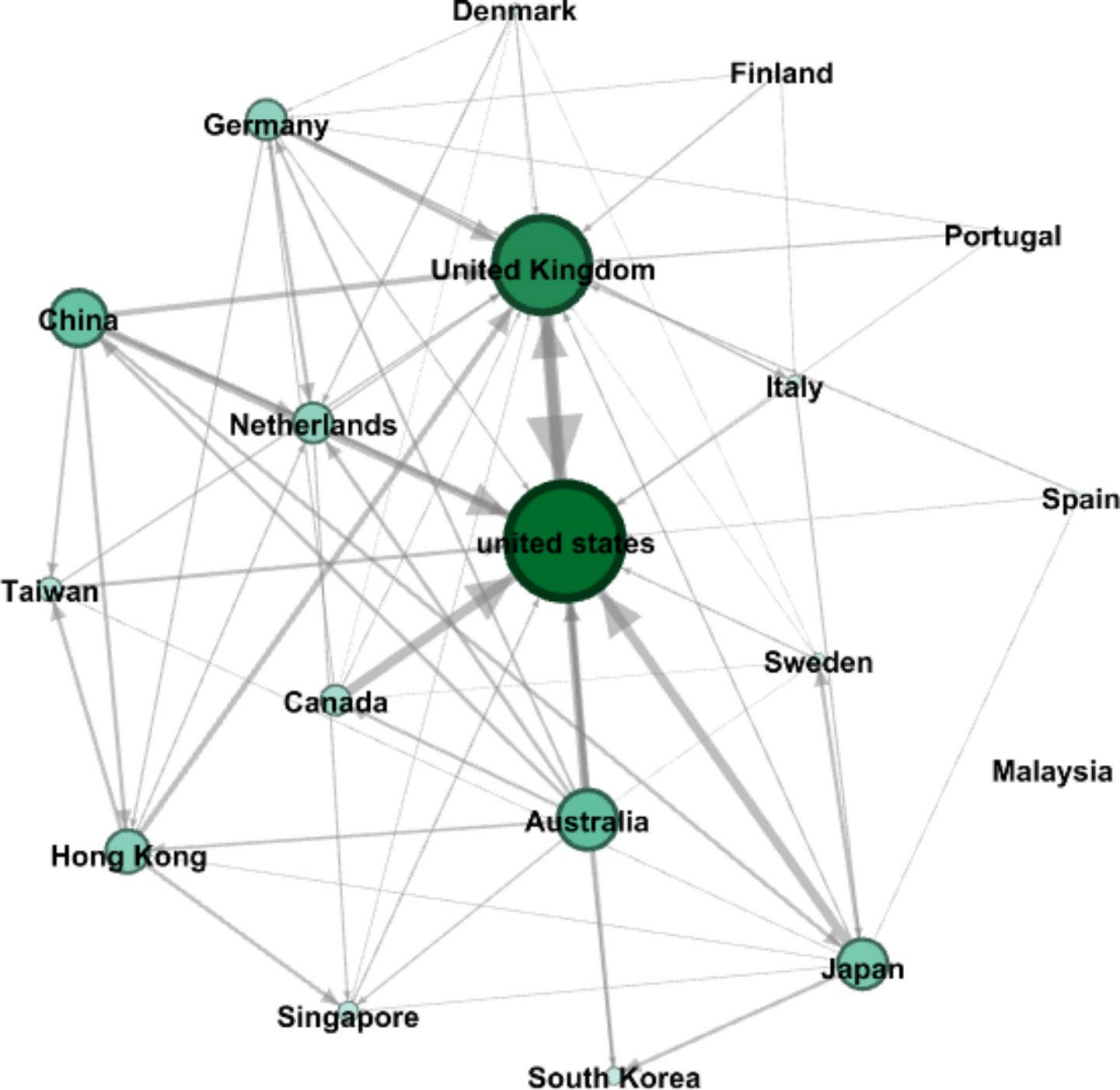
Prominent countries producing research outputs on the APS nexus

In *Gephi*, the resultant network map is represented as six nodes linked together by six links, with the darker colour denoting a node with a higher weighted degree score. Thus, it is easy to see how much impact each country has by looking at their weighted degrees. As shown in Fig. 6, the USA is the most influential country, with significant ties to the United Kingdom. Regarding research collaboration in the APS nexus, another important connection exists between Canada and the USA; Japan and the USA; Netherland and the USA. It also demonstrates the growing number of studies in the Asia Pacific, including Australia, China, Hong Kong, Japan, Malaysia, Singapore, South Korea, and Taiwan. While South American and African countries do not feature on the collaboration network map, European countries like Netherland, Portugal, Germany, Finland, Italy, and Sweden appear connected to international collaborative networks.

## Conclusion, implications, and limitations

The scientometric analysis conducted in this study analysed 566 APS-related journal articles published between 2011 and 2020 utilising the Scopus database. In line with the contention that “bridging the gap between research and policy is a constant challenge in academia despite broad acknowledgment that it is crucial for informing policymaking with scientific evidence” (Sharifi et al. 2021, p. 664), the main contribution of this paper is highlighting the fact that such scientometric analysis is not only suited for examining various elements of current research tends but also inform policy discussions and guide future research directions.

On the one hand, the past decade did witness an increase in the number of journal articles published. On the other, it is clear that the literature on the APS nexus remains fragmented due to its multidisciplinary nature. The top ten influential research topics were ageing, older people, health, demography, older adults, gender, population ageing, ageing society, active ageing, and quality of life. The analysis revealed less attention had been paid to contextually important policy matters topics such as sustainable development, social policy, public health, health policy, disability, and caregiving. Although the analysis revealed two main collaborative networks, not all influential authors were members of the established collaborative networks. The analysis clearly showed that developed economies in the OECD block, such as Australia, the UK, and the US stand out as significant countries producing and collaborating in APS-related studies. Although the challenges associated with the APS nexus have become a global priority, especially in the context of SDGs in developing countries (WHO, 2019), the findings concur with the trend that research outputs in developing countries have not been encouraging (see Dhakal and Aryal 2022). With the rapidly growing ageing population and associated societal challenges, the APS nexus will continue to gain currency in the future. Two implications of the analysis: (a) policy insights and future research direction, and (b) north-south collaboration are discussed below.

### Policy insights and future research direction

First, the trend of key research topics presented above aligns with the priorities identified by the *Research Agenda on Ageing for the Twenty-First Century* (United Nations, 2007). However, policy themes relevant to the futuristic context, such as disability, health policy, public health, social policy, and sustainable development, have received less attention. Future studies should consider these shortcomings (see Mahmood & Dhakal, 2022) and build on the analysis presented in the paper to examine under-represented research themes. Second, findings also indicate that the policy-focused research on the APS nexus remains scarce. This finding is not unique to the APS nexus, as similar trends are common in other social science disciplines (Virani et al. 2020). In the context of an ongoing COVD-19 pandemic, various aging-related policy issues are likely to emerge as new research priorities in the immediate future (Dhakal et al. 2021; Solanki et al., 2021). As the primary purpose of social policy innovation is to improve human wellbeing, especially the welfare of those disadvantaged, marginalised, and vulnerable (see Dhakal & Burgess, 2021), future studies should consider these observations to inform evidence-based policy discussions (see Head 2016) and guide future research directions.

### North-south research collaboration

First, the rise of APS-related research outputs in the developed countries, while a welcoming trend, also suggests that the developing nations have been left out. The analysis clearly showed that developed economies in the OECD block, such as Australia, the UK, and the US stand out as significant countries producing and collaborating in APS-related studies. There have been some laudable but isolated efforts to examine the APS-related literature in developing countries (see Cheema 2013; Nguyen et al. 2019; Dhakal and Aryal 2022; Chatterjee and Mahmood 2022). However, Mahmood & Dhakal (2022) point out that while Japan is the front runner in research outputs amongst countries in the Asia Pacific region, collaborative outcomes from developing countries on the APS nexus have been negligible. For instance, although European Union-funded projects have been demanding research outputs be applied and achieve policy outcomes for decades (see Ettore, 2000), the north-south research collaborations are not necessarily an easy undertaking (Bradley 2008; Syed et al. 2012; Bleck et al. 2018). In the specific context of SDG # 17 – revitalize the global partnership for sustainable development (United Nations, 2020), researchers and research institutions should take these findings into account as a justification to build and maintain research collaboration between north and south in terms of agenda-setting for the APS-related policy innovation.

### Limitations

As with any scientific examination, there are limitations to the analysis presented in this paper. The scientometric analytical approaches, when used efficiently, could yield significant research outcomes through the effective combination of qualitative, quantitative, and state-of-the-art computational methods. However, factored by the critical challenges associated with the usage of the technique, such as the over-emphasis on the keyword searches, the aspect of limited datasets to extract information from, and the inadequacy of importance laid on the recency of publications studies reflect critical views on the quality of visual network-maps. The same concerns reflect on the selection of nodes and their connections. In addition, the analysis was limited to the years between 2011 and 2020. Notwithstanding the impact of COVID-19 pandemic on all sectors of society (Dhakal et al., 2021) and its significance on the ageing-related research and policy agenda (see Xie et al. 2021; Miller, 2021), articles that examined the APS nexus with a lens of the pandemic were not part of the current analysis.
